# Transcutaneous auricular vagus nerve stimulation improves working memory in temporal lobe epilepsy: A randomized double‐blind study

**DOI:** 10.1111/cns.14395

**Published:** 2023-08-08

**Authors:** Liping Pan, Jiajing Wang, Wenjuan Wu, Yunfan Wang, Yujing Zhu, Yijun Song

**Affiliations:** ^1^ General Medicine Department Tianjin Medical University General Hospital Tianjin China; ^2^ Department of Intensive Care Medicine, State Key Laboratory of Experimental Hematology, National Clinical Research Center for Blood Diseases, Haihe Laboratory of Cell Ecosystem, Institute of Hematology and Blood Diseases Hospital Chinese Academy of Medical Sciences and Peking Union Medical College Tianjin China; ^3^ Tianjin Medical University Tianjin China; ^4^ Department of Neurology The First Affiliated Hospital of Henan University of Science and Technology Luoyang China

**Keywords:** frontal midline theta, neural oscillation, refractory temporal lobe epilepsy, transcutaneous vagus nerve stimulation, working memory

## Abstract

**Aims:**

This study investigated the impact of transcutaneous auricular vagus nerve stimulation (taVNS) on working memory (WM) in refractory temporal lobe epilepsy (rTLE) and the underlying mechanisms.

**Methods:**

In this randomized double‐blind study, 28 rTLE patients were subjected to an active or sham taVNS (a/s‐taVNS) protocol for 20 weeks (a‐taVNS group, *n* = 19; s‐ta VNS group, *n* = 9). Patients performed visual WM tasks during stimulation and neural oscillations were simultaneously recorded by 19‐channel electroencephalography.

**Results:**

Compared with the baseline state, reaction time was significantly shorter after 20 weeks of taVNS in the a‐taVNS group (*p* = 0.010), whereas no difference was observed in the s‐taVNS group (*p* > 0.05). The power spectral density (PSD) of the theta frequency band in the Fz channel decreased significantly after a‐taVNS during WM‐encoding (*p* = 0.020), maintenance (*p* = 0.038), and retrieval (*p* = 0.039) phases, but not in the s‐taVNS group (all *p* > 0.05).

**Conclusion:**

Neural oscillations during WM were altered by taVNS and WM performance was improved. Alterations in frontal midline theta oscillations may be a marker for the effect of taVNS on cognitive regulation.

## Graphical abstract

In the randomized, double‐blind study, neural oscillations during WM were altered and WM performance was improved after taVNS treatment.

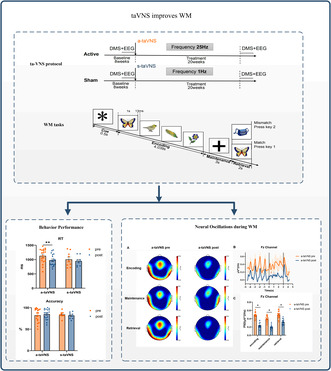

## 1 INTRODUCTION

Working memory (WM) is a core component of higher‐level cognition functions, which constitute a system that combines attention control with temporary storage and information processing.^1^, ^2^ WM consists of encoding, maintenance, and retrieval phases.^3^ WM impairment is a feature of many neurologic and neuropsychiatric disorders such as attention deficit hyperactivity disorder^3^, ^4^ and epilepsy,^5^, ^6^ and has no effective treatment.

Vagus nerve stimulation (VNS) is widely used in the treatment of epilepsy^7^ and has been shown to improve cognitive function, including WM,^8^, ^9^ attentional processing,^10^ and associative memory.^11^ Transcutaneous auricular VNS (taVNS) is a non‐invasive approach for cranial nerve stimulation and is a promising treatment for improving WM performance.^12^, ^13^ Task accuracy was found to be improved in the post‐taVNS state in healthy subjects performing a WM task.^8^ A longitudinal study with respect to the influence of long‐term taVNS on cognitive function showed that taVNS did not improve verbal memory performance in patients with drug‐resistant epilepsy, while immediate recall and delayed recognition scores improved significantly after 6 weeks of acute invasive VNS treatment, suggesting that longer, repeated stimulation of the vagal pathway is effective.^14^ The impact of chronic taVNS on WM in patients with epilepsy and the underlying mechanisms are unknown.

Neural oscillations are involved in information encoding and transmission in WM.^15^ Theta activity is responsible for integrating top‐down and bottom‐up information processing and the allocation of cognitive resources.^16^ We previously demonstrated that the power of the theta frequency band was significantly higher than that of other frequency bands during a WM task and was higher in the frontal region than in other brain regions.^17^ Frontal theta power was found to be significantly lower in temporal lobe epilepsy (TLE) patients with WM impairment than in normal subjects during the WM encoding, maintenance, and retrieval phases; moreover, reduced frontal theta power during the encoding phase was associated with longer reaction times (RTs).^17^ VNS increased N1 amplitude and frontal alpha asymmetry during WM tasks and decreased error rates in patients with refractory epilepsy.^18^ In healthy subjects subjected to acute active/sham taVNS during the Go/NoGo task, peak N2 amplitude in the frontal region was decreased as determined based on electroencephalography (EEG) event‐related potentials.^10^ Taken together, these studies suggest that VNS preferentially modulates task‐related neural oscillations and is thus a more sensitive indicator of cognition than behavioral test performance.

Patients with refractory TLE (rTLE) often have WM deficits with associated alterations in neural oscillation.^17^, ^19^, ^20^ In this study, we examined the effects of chronic taVNS on WM in rTLE patients by performing EEG recordings during the encoding, maintenance, and retrieval phase of WM and analyzing changes in neural oscillations in the WM task state.

## 2 METHODS

### 2.1 Participants

A total of 30 patients with drug refractory TLE (rTLE) were recruited at Tianjin Medical University General Hospital. Inclusion criteria were as follows: (1) patients with rTLE as defined by the International League Against Epilepsy (2010 version)^21^, ^22^; (2) diagnosed with TLE based on seizure symptomatology, 24‐h video EEG monitoring, and magnetic resonance imaging findings; (3) right‐handed; and (4) voluntarily signed the informed consent form and cooperated with the testing. Exclusion criteria were as follows: (1) a history of severe neurologic, mental, or systemic diseases; (2) drug or alcohol abuse; (3) women who were pregnant or lactating; and (4) patients with a pacemaker, vagus stimulator, or metal implant in the body. Participants were randomly divided into active and sham taVNS groups (a‐taVNS group, *n* = 20; s‐taVNS group, *n* = 10) in a 2:1 ratio. All subjects provided written, informed consent before participating in the study, which was approved by the Ethics Committee of Tianjin Medical University General Hospital.

### 2.2 VNS protocol

**FIGURE 1:**
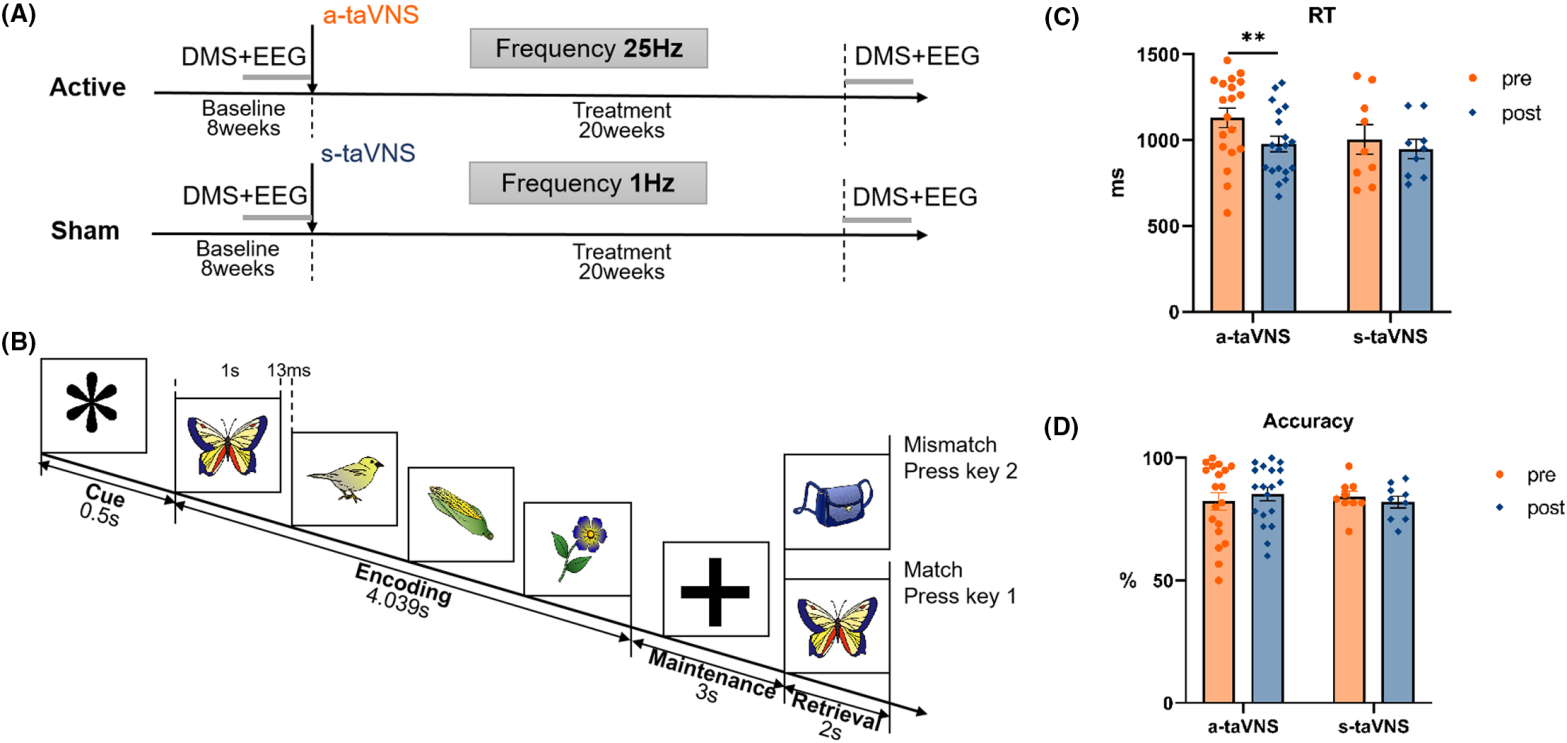
Experimental design and behavioral performance. (A) taVNS treatment protocol. The 8‐week baseline period was followed by a 20‐week a‐/s‐taVNS treatment period (taVNS frequencies of 25 and 1 Hz, respectively). Subjects received taVNS 3–5 times/day for 30 min each. (B) Visual WM paradigm. A 0.5‐s gaze point (“*”) was shown at the beginning of each trial, followed by the encoding phase during which four images were presented in sequence, each lasting 1 s and separated by 13 ms. After the fourth image disappeared, a “+” was shown to indicate the beginning of the 3‐s maintenance phase. A probe image was then presented in the retrieval phase, and subjects were required to determine whether the probe image matched one of the images in the memory set within 2 s. (C) RT in the WM task. Compared with the pre‐taVNS state, RT was significantly shorter in the post‐taVNS state in the a‐taVNS group, whereas no significant difference in RT was observed between the pre‐ and post‐taVNS states in the s‐taVNS group. (D) Accuracy in the WM task. There was no significant difference in accuracy between pre‐ and post‐taVNS states in the a‐taVNS and s‐taVNS groups. ACC, accuracy; DMS, delayed matching‐to‐sample; RT, reaction time; taVNS, transcutaneous auricular vagus nerve stimulation; WM, working memory. **p < 0.01.

We used a randomized, double‐blinded, within‐subject study design. An 8‐week baseline period preceded taVNS and patients did not receive any treatment besides antiseizure medications, during which seizure frequency was recorded. During the 20‐week a‐/s‐taVNS treatment period, stimulation was delivered using a taVNS device (Jiangxi Xinzhile Medical Technology Co; TVNS‐100) comprising two auricular electrodes connected to a control unit. The stimulating electrode was placed at the cymba conchae, which is heavily innervated by the auricular branch of the vagus nerve.^23^ The active and sham taVNS frequencies were 25 and 1 Hz, respectively (Figure 1A). taVNS was performed 3–5 times a day for 30 min each. The stimulus intensity was set to the maximum value acceptable to subjects (range: 30–50 V), with a pulse width of 250 μs.

Subjects were not informed of the type of stimulus (active vs. sham) or expected outcome. Two subjects (one in each group) withdrew from the study during taVNS treatment.

### 2.3 WM task

Subjects performed a visual delayed matching‐to‐sample (DMS) task (Figure 1B) at the end of the baseline period before the first taVNS treatment and after the last treatment. Using images of objects from the Snodgrass image library^24^ as visual stimuli, a 0.5‐s gaze point “*” was shown in the center of the screen at the beginning of each trial; the encoding phase began when the “*” disappeared. Four images were presented in sequence, each lasting 1 s and separated by 13 ms. After the fourth image disappeared, a “+” was shown on the screen to remind the participant of the 3‐s maintenance phase. A probe image was then presented in the retrieval phase, and subjects had to decide whether the probe image matched one of the images in the memory set within 2 s. The test included 6 blocks of the task with 10 trials each. Task performance was measured by accuracy and mean reaction time (RT) of correct trials.

### 2.4 EEG recording and data preprocessing

Scalp EEG signals (19 channels) were recorded during the DMS task using a Nicolet One EEG system (Natus Medical) with a sampling frequency of 1024 Hz and impedance <5 kΩ. Scalp electrodes (Fp1, Fp2, F7, F3, Fz, F4, F8, T3, C3, Cz, C4, T4, T5, P3, Pz, P4, T6, O1, and O2) were positioned according to the International 10/20 system. The reference electrode was placed near Cz, and the ground electrode was placed on the forehead. The EEG signal was low pass‐filtered (100 Hz), notch‐filtered (49–51 Hz), and re‐referenced to the common average reference value. Eye movement and electromyography artifacts were removed from the original signals using the EEGLAB toolbox, and baseline drift was removed in MATLAB v2012b (MathWorks).

### 2.5 Analysis of neural oscillation pattern during WM phases

Short‐time Fourier transform (STFT) was applied to EEG components in delta‐ (0.05–4 Hz), theta‐ (4–8 Hz), alpha‐ (8–13 Hz), beta‐ (13–30 Hz), and gamma (30–100 Hz)‐frequency bands to calculate power spectral density (PSD) during each WM phase using a 0.4‐s–wide Hamming window and 0.5‐Hz frequency smoothing.^25^ STFT was calculated as follows:

(1) $$\text{STFT}\left(f,t\right)=\int_{-\infty }^{+\infty }\left[x\left(t\right)g\left(t-\tau \right)\right]e^{-j2\mathrm{\pi f\tau}}\mathrm{d\tau}$$

where *x*(*t*) represents the EEG signal and *g*(*t*) represents a window function. PSD values of different frequency bands were separately calculated for the encoding, maintenance, and retrieval phases, and the frequency band with the maximum PSD was defined as the prominent frequency band of each WM phase and retained for subsequent analysis.

In order to determine the change in EEG power related to WM, the PSD of WM efficiency (*P*_wm_) was calculated as follows:

(2) $$P_{\mathrm{wm}}=P_{\mathrm{raw}}-P_{\mathrm{rs}}$$

where *P*_raw_ is the PSD of neural oscillations recorded during the WM task and *P*_rs_ is the baseline PSD recorded in the resting state (i.e., 5 min with eyes closed before the start of the WM task).

EEG power of each WM phase was defined based on a previous study.^17^ Briefly, EEG power during the encoding phase was defined as the average power of four peaks (100 ms around each peak) during encoding around the time the sequence images were shown; EEG power during the maintenance phase was defined as the average EEG power of the 3 s maintenance phase; and EEG power during the retrieval phase was defined as the average power between the two peaks after the appearance of the probe image (Figure S1).

In order to identify prominent brain region(s) involved in WM, EEG channels were grouped into five clusters: frontal region (Fp1, Fp2, F7, F3, Fz, F4, and F8); central‐parietal region (C3, Cz, C4, P3, Pz, and P4); occipital region (O1 and O2); left temporal region (T3 and T5); and right temporal region (T4 and T6). The PSD of the prominent frequency band in each brain region during the WM task before taVNS stimulation was calculated and the brain region with the highest PSD was defined as the prominent brain region. The spatial distribution of PSD in the 19 channels of the prominent frequency band during WM encoding, maintenance, and retrieval phases was plotted separately, and the spatial distribution patterns before and after taVNS were compared to determine the effect of taVNS on neural oscillations during each phase.

### 2.6 Statistical analysis

Data are expressed as mean ± standard error of the mean unless otherwise stated, and data normality was confirmed with the Shapiro–Wilk test (*p* > 0.05). The *t*‐test was used to assess the statistical significance of differences between groups, and Levene's test was used to assess the homogeneity of error variances between groups. The chi‐squared test was used to analyze dichotomous variables. One‐way analysis of variance was used to compare data in more than three groups with the least significant difference test used for post hoc analysis. Pearson's correlation analysis was used to evaluate the relationship between neural oscillation and WM task performance. All statistical analyses were performed using SPSS v25.0 (IBM), and the significance level was set at *α* = 0.05 (*p* < 0.05).

## 3 RESULTS

### 3.1 Behavior test performance

The demographic and clinical characteristics of subjects are shown in Table 1. Comparison of WM task performance before and after taVNS showed that compared with the pre‐taVNS state, RT was significantly reduced after taVNS in the a‐taVNS group (pre‐taVNS vs. post‐taVNS:1085.72 vs. 961.50 ms, *p* = 0.010) (Figure 1C,D). There was no significant difference in accuracy before vs. after stimulation in the a‐taVNS group (pre‐taVNS vs. post‐taVNS: 82.5% vs. 85.5%, *p* = 0.116). In the s‐taVNS group, there was no difference in WM performance before versus after taVNS (accuracy pre‐taVNS vs. post‐taVNS: 78.8% vs. 82.1%, *p* = 0.730; RT pre‐taVNS vs. post‐taVNS: 996.64 vs. 917.09 ms, *p* = 0.134).

**TABLE 1:** Demographic and clinical characteristics of subjects.

	a‐taVNS (*n* = 19)	s‐taVNS (*n* = 9)	*p*‐Value
Age (years)	36.95 ± 11.63	42.00 ± 9.67	0.299^a^
Male/Female	15/4	7/2	0.944^b^
Education (years)	12.74 ± 3.21	11.82 ± 2.79	0.851^a^
Disease duration (years)	15.79 ± 13.41	15.09 ± 8.08	0.580^a^
Seizure frequency (per month)	5.17 ± 3.06	5.91 ± 3.40	0.393^a^
ASMs species	2.53 ± 0.50	2.82 ± 0.57	0.090^a^
MMSE score	27.20 ± 2.79	28.20 ± 2.14	0.449^a^
MoCA score	20.89 ± 5.67	21.82 ± 4.45	0.519^a^

*Note*: Values are mean and standard deviation.

Abbreviations: ASMs, antiseizure medications; MMSE, mini‐mental state examination; MoCA, Montreal Cognitive Assessment.

^a^ *t*‐test.

^b^ Chi‐squared test.

### 3.2 Neural oscillation patterns in the pre‐taVNS state during the WM task

**FIGURE 2:**
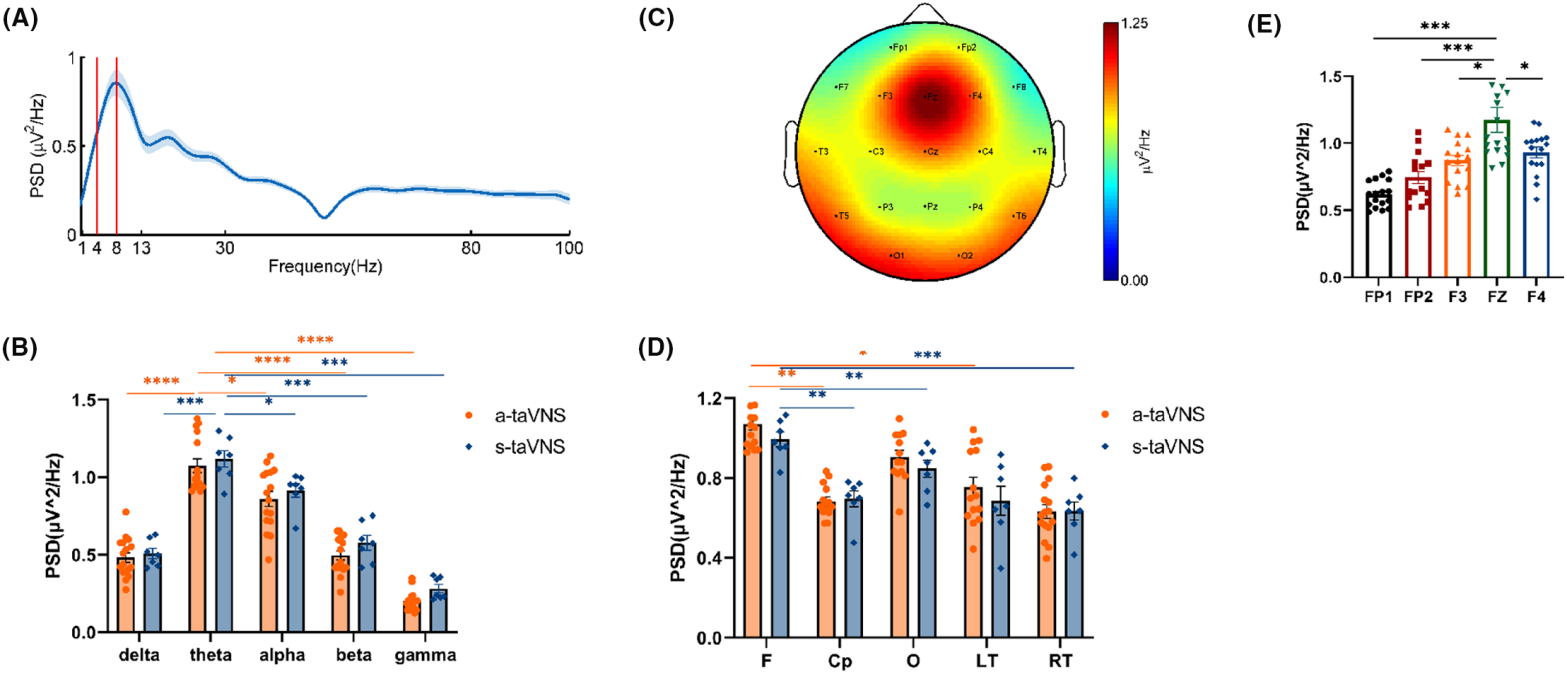
Distribution of EEG oscillations during the WM task. (A) Average PSD curve as a function of frequency during WM. The *x*‐axis indicates frequency, and the area between the two solid red lines indicate the theta‐frequency range. The *y*‐axis shows the average PSD of 19 channels during the WM task. (B) Comparison of average PSD values among delta, theta, alpha, beta, and gamma frequencies during WM. The *x*‐axis indicates the frequency bands, and the *y*‐axis indicates the average PSD of 19 channels for each frequency band. The PSD of the theta‐frequency band was significantly higher than that of the delta, alpha, beta, and gamma bands. There was no significant difference in average PSD of each frequency band between the a‐taVNS and s‐taVNS groups. (C) Spectral distribution of theta oscillation in all subjects. Power is indicated by color. (D) Histogram of average theta PSD in different brain regions. The average theta PSD was higher in the frontal and occipital regions than in other brain regions, and there was no significant difference in theta PSD in each brain region between a‐taVNS and s‐taVNS groups. (E) Histogram of theta PSD in different channels in the frontal region in all subjects. Theta PSD was higher in the Fz channel than in other channels. Cp, central‐parietal region; F, frontal region; Lt, left temporal region; O, occipital region; Rt, right temporal region. *p < 0.05; **p < 0.01; ***p < 0.001; and ****p < 0.0001.

Neural oscillation patterns in all subjects in the pre‐taVNS state during the WM task are shown in Figure 2. Theta oscillations were observed during the WM task (Figure 2A). There were significant differences in the PSD of delta‐, theta‐, alpha‐, beta‐, and gamma‐frequency bands (a‐taVNS group: *F* = 54.32, *p* < 0.001; s‐taVNS group: *F* = 63.30, *p* < 0.001) (Figure 2B). The PSD of the theta frequency band was significantly higher than that of other frequency bands both in the a‐taVNS group (theta vs. delta, *p* < 0.001; theta vs. alpha, *p* = 0.026; theta vs. beta, *p* < 0.001; theta vs. gamma, *p* < 0.001) and s‐taVNS group (theta vs. alpha, *p* = 0.014; theta vs. others, *p* < 0.001). There was no significant difference in the PSD of each frequency band between a‐taVNS and s‐taVNS groups (delta, *p* = 0.613; theta, *p* = 0.546; alpha, *p* = 0.496; beta, *p* = 0.804; gamma, *p* = 0.28). Thus, the theta‐frequency band was considered the most prominent during the WM task. In terms of the distribution of neural oscillations, there were significant differences in theta PSD among the frontal, central‐parietal, occipital, left temporal, and right temporal regions (a‐taVNS group: *F* = 6.154, *p* < 0.001; s‐taVNS group: *F* = 8.434, *p* < 0.001) (Figure 2C,D). Theta PSD was significantly higher in the frontal region than in the central‐parietal (a‐taVNS group, *p* = 0.009; s‐taVNS group, *p* = 0.004), left temporal (a‐taVNS group, *p* = 0.014; s‐taVNS group, *p* = 0.003), and right temporal (a‐taVNS group, *p* = 0.001; s‐taVNS group, *p* < 0.001) regions. There was no significant difference in theta PSD between the frontal and occipital regions (a‐taVNS group, *p* = 0.993; s‐taVNS group, *p* = 0.738). Theta PSD was higher in the occipital region than in the right temporal region (a‐taVNS group, *p* = 0.028; s‐taVNS group, *p* = 0.012), whereas no difference was observed between the occipital and other regions (a‐taVNS group: O vs. Cp, *p* = 0.054; O vs. Lt, *p* = 0.063; s‐taVNS group: O vs. Cp, *p* = 0.079; O vs. Lt, *p* = 0.059). Thus, the frontal region was considered the prominent brain region during the WM task. Theta PSD in frontal (*p* = 0.681), central‐parietal (*p* = 0.510), occipital (*p* = 0.233), left temporal (*p* = 0.432), and right temporal regions (*p* = 0.396) did not differ between the a‐taVNS and s‐taVNS groups.

In the frontal region, theta PSD differed among Fp1, Fp2, F3, Fz, and F4 channels (*F* = 9.074, *p* < 0.001), with theta PSD higher in the Fz channel than in the other channels (Fz vs. Fp1, *p* < 0.001; Fz vs. Fp2, *p* < 0.001; Fz vs. F3, *p* = 0.017; Fz vs. F4, *p* = 0.035) (Figure 2E). The Fz channel was therefore considered the most prominent channel during the WM task.

**FIGURE 3:**
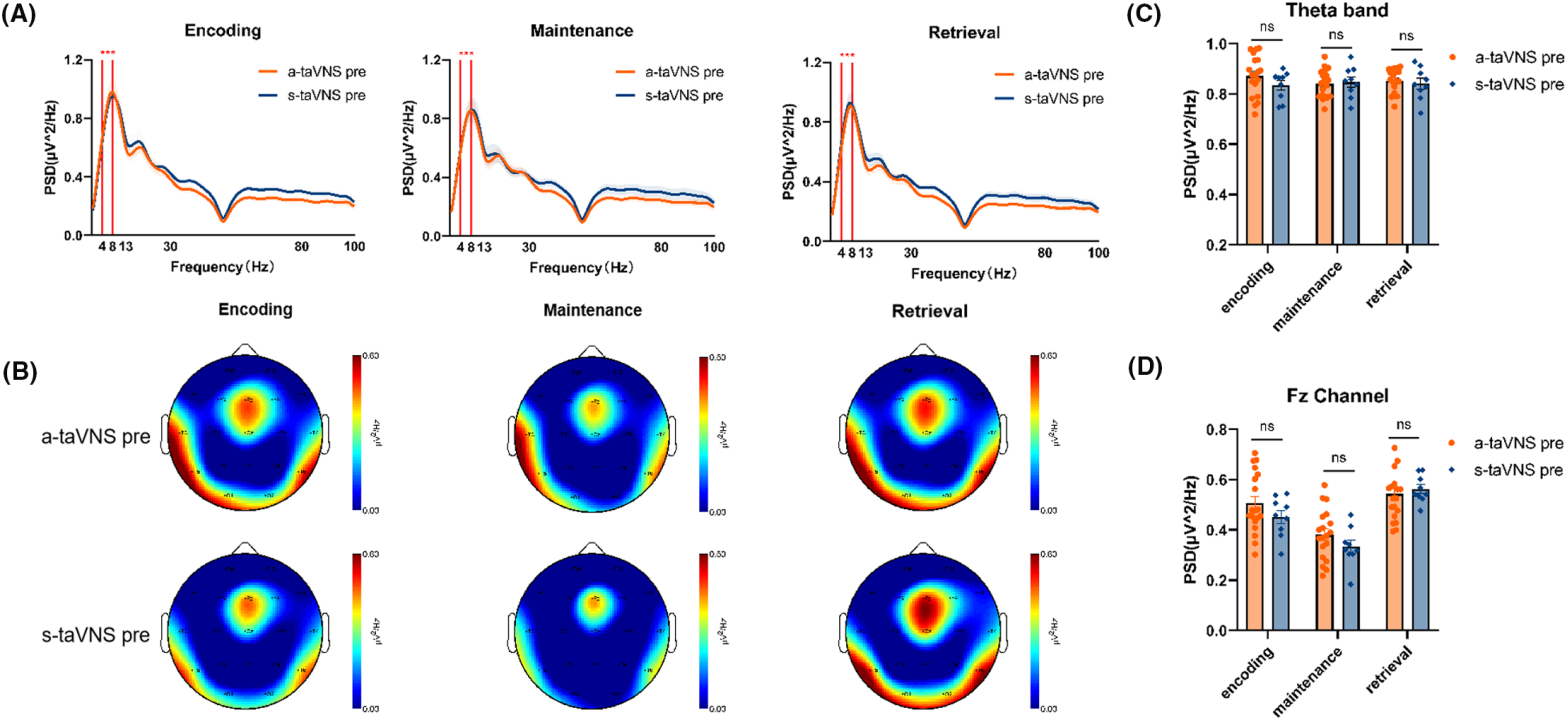
Patterns of neural oscillation during each WM phase in the pre‐taVNS state. (A) Average PSD curve as a function of frequency during WM‐encoding, maintenance, and retrieval phases. The theta‐frequency band was prominent during each phase. (B) Spatial distribution of theta oscillations during each WM phase. Power is indicated by color. Theta PSD was concentrated in the frontal region, and the Fz channel was prominent. (C and D) Comparison of theta PSD in the whole brain (C) and Fz channel (D) between the a‐taVNS and s‐taVNS groups during each WM phase. There was no significant difference between the two groups in any WM phase before taVNS treatment. ns, non‐significant.

Patterns of neural oscillations in subjects were assessed during each WM phase in the pre‐taVNS state (Figure 3). The PSD of the theta frequency band was higher than that of the delta‐, alpha‐, beta‐, and gamma‐frequency bands in the encoding, maintenance, and retrieval phases of WM (Figure 3A); theta PSD was mainly concentrated in the frontal region, especially the Fz channel (encoding: *F* = 10.85, *p* < 0.001; Fz vs. F3, *p* = 0.003; Fz vs. F4, *p* = 0.009; Fz vs. FP1, *p* < 0.001; Fz vs. FP2, *p* < 0.001; maintenance: *F* = 7.953, *p* < 0.001; Fz vs. F3, *p* = 0.012; Fz vs. F4, *p* = 0.032; Fz vs. FP1, *p* < 0.001; Fz vs. FP2, *p* < 0.001; and retrieval: *F* = 9.330, *p* < 0.001; Fz vs. F3, *p* = 0.002; Fz vs. F4, *p* = 0.008; Fz vs. FP1, *p* < 0.001; Fz vs. FP2, *p* < 0.001) (Figure 3B). There were no significant differences in theta PSD in the whole brain between a‐taVNS and s‐taVNS groups (encoding *t* = 0.438, *p* = 0.667; maintenance *t* = 0.076, *p* = 0.940; retrieval *t* = 0.590, *p* = 0.563) (Figure 3C), and no significant differences in theta PSD in the Fz channel between groups in the pre‐taVNS state during W‐encoding (*t* = 0.416, *p* = 0.682), maintenance (*t* = 0.814, *p* = 0.42), and retrieval (*t* = 0.150, *p* = 0.882) phases (Figure 3D).

### 3.3 Patterns of neural oscillation during each WM phase after taVNS

**FIGURE 4:**
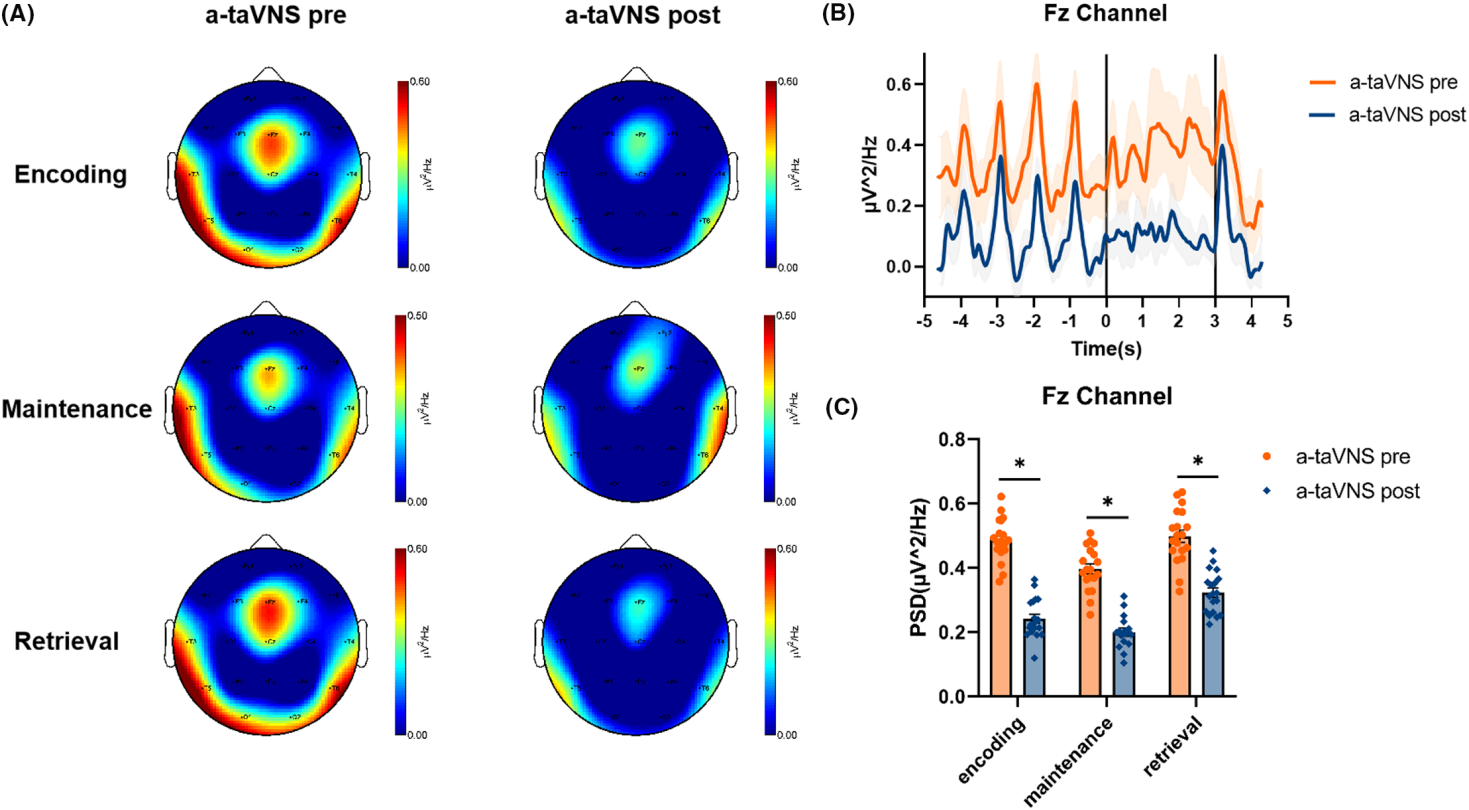
Characteristic theta oscillation patterns in the a‐taVNS group during each WM phase. (A) Spectral distribution of theta power in the pre‐taVNS state and the post‐taVNS state during the encoding, maintenance, and retrieval phases of WM. (B) Representative plot of average theta PSD in Fz channel during the WM task. The *x*‐axis shows the time course of the task; the left, central, and right areas separated by the solid black lines represent the encoding, maintenance, and retrieval phases of WM, respectively. The *y*‐axis shows the average theta PSD in the Fz channel. (C) Comparison of theta PSDs in the Fz channel between the pre‐ and post‐taVNS states during each WM phase. The theta PSD was reduced in the post‐taVNS state compared with the pre‐taVNS state. *p < 0.05.

**FIGURE 5:**
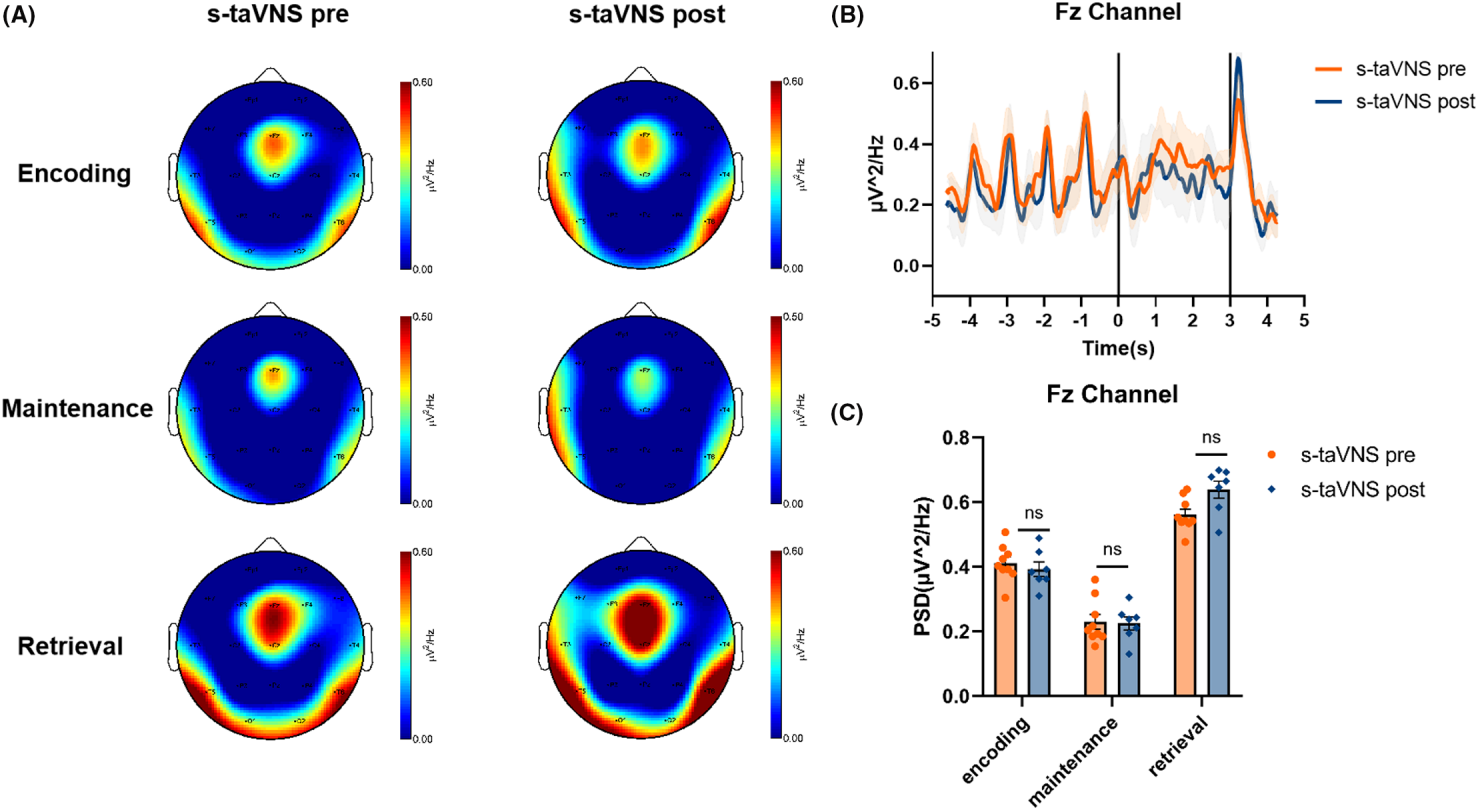
Characteristic patterns of theta oscillation in the s‐taVNS groups during each WM phase. (A) Spectral distribution of theta power in the pre‐ and post‐taVNS states during the WM‐encoding, maintenance, and retrieval phases. (B) Representative plot of average theta PSD in Fz channel during the WM task. The *x*‐axis shows the time course of the task; the left, central, and right areas separated by the solid black lines represent the encoding, maintenance, and retrieval phases of WM, respectively. The *y*‐axis shows the average theta PSD in the Fz channel during the WM task. (C) Comparison of theta PSDs in Fz channel in the pre‐ versus post‐taVNS states during each WM phase. There was no significant difference in theta PSD between the two states.

The spectral distribution of theta PSD in the pre‐ and post‐taVNS states during each WM phase in the a‐taVNS group is shown in Figure 4. Compared with the pre‐stimulation state, theta PSD in the Fz channel was significantly lower in the post‐taVNS state during WM encoding (*t* = 2.473, *p* = 0.020), maintenance (*t* = 2.172, *p* = 0.038), and retrieval (*t* = 2.168, *p* = 0.039) phases. We compared the spectral distribution of theta PSD in the pre‐ and the post‐taVNS states during each WM phase in the s‐taVNS group and found no significant difference in theta PSD in the Fz channel between pre‐ and post‐taVNS states in the s‐taVNS group during the WM‐encoding (*t* = 0.256, *p* = 0.803), maintenance (*t* = 0.350, *p* = 0.733), and retrieval (*t* = 1.082, *p* = 0.302) phases (Figure 5).

### 3.4 Correlation analysis between theta oscillations and memory test performance

**FIGURE 6:**
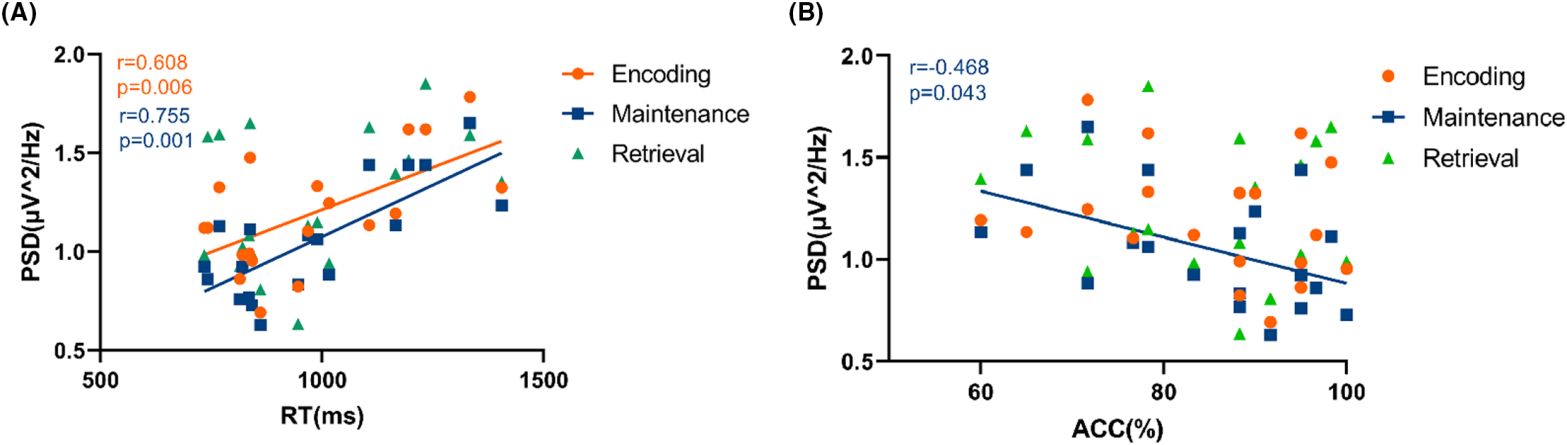
Correlation between theta PSD in the Fz channel in the post‐taVNS state during each WM phase and performance in the a‐taVNS group. (A) Correlation between theta PSD and RT during each WM phase. (B) Correlation between theta PSD and ACC during each WM phase.

The above results indicated that theta PSD in the Fz channel altered after stimulation in the a‐taVNS group. We therefore conducted a correlation analysis to clarify the relation between the altered theta oscillations in the Fz channel in the post‐taVNS state during each WM phase and memory test performance in the a‐taVNS group (Figure 6). Theta PSD was positively correlated with RT during the encoding phase (*r* = 0.608, *p* = 0.006) and maintenance phase (*r* = 0.755, *p* = 0.001), but was unrelated to RT during the retrieval phases (*r* = 0.402, *p* = 0.088) (Figure 6A). A negative correlation was identified between theta PSD and ACC during the maintenance (*r* = −0.468, *p* = 0.043), whereas no correlations were observed between theta PSD and ACC during the encoding phase (*r* = −0.265, *p* = 0.272) and retrieval phase (*r* = −0.214, *p* = 0.378) (Figure 6B).

## 4 DISCUSSION

In this study, neural oscillations during each WM phase in the pre‐ and post‐taVNS states were analyzed to explore the impact of taVNS on WM in rTLE patients. Our results showed that active taVNS treatment improved WM behavior performance after 20 weeks, mainly by reducing the RT required for the task, and theta PSD in the Fz channel was reduced during the encoding, maintenance, and retrieval phases of WM. No similar effects were observed in the sham taVNS group.

Frontal midline theta (FMT) are theta oscillations recorded near the Fz channel that plays an important role in WM.^26^, ^27^ In the current study, FMT power was increased during the WM encoding, maintenance, and retrieval phases, as reported in previous studies.^28^, ^29^, ^30^ Source modeling based on scalp EEG and magnetoencephalography data suggests that FMT may be generated by sources in the anterior cingulate and medial prefrontal cortex (PFC).^31^ During the encoding phase, the frontal region receives projections from the hippocampus, and FMT regulates the gating system and mediates the sequential encoding of information.^32^, ^33^, ^34^ In the maintenance phase, FMT maintains the storage of task‐relevant information by regulating posterior cortical activity through top‐down control^26^, ^35^, ^36^ and mediates cognitive control functions.^37^ During the retrieval phase, the high power of frontal midline theta is associated with memory retrieval, reflecting task decision‐making.^30^, ^38^ Furthermore, FMT increases with WM load, task difficulty, and cognitive effort,^39^, ^40^, ^41^ and alterations in FMT can predict WM behavior performance.^26^, ^27^, ^42^ In our study, compared with the pre‐taVNS state, theta PSD decreased in the post‐taVNS state, suggesting that subjects in the post‐taVNS state require less cognitive effort to perform the same task.

Frontal midline theta activity associated with WM is regulated by the norepinephrine (NE) and γ‐aminobutyric acid (GABA) systems.^43^, ^44^ The concept of WM gating has been widely used to describe the mechanism by which WM can flexibly switch between maintenance and updating.^45^ As opposed to gate closing, gate opening represents a switch from WM maintenance to updating, reflecting active information encoding and decision‐making.^46^ Prefrontal theta activity is known to be closely related to WM gating and strongly modulated by the NE system.^47^, ^48^ Using the reference‐back paradigm, Yu et al.^34^ recorded EEG signals and tracked pupil diameter as an indirect measure of NE system activity. They found modulation of the dorsolateral prefrontal theta band synchronization process during gate opening and a strong correlation between theta synchronization and pupil diameter, suggesting an important role of the NE system in the time‐on‐task effects on WM gate opening. Mid‐frontal and suprafrontal theta oscillations, which are known to reflect response selection and control processes associated with task maintenance,^16^, ^49^, ^50^ are regulated through gain adjustment mechanisms in the NE system.^51^, ^52^, ^53^ NE improves the signal‐to‐noise ratio in neural circuits by modulating the ability of the neural network to distinguish between relevant and irrelevant information.^51^, ^54^ Adelhöfer et al.^55^ found a significant positive correlation between the superior frontal gyrus theta band activity and pupil diameter time courses in healthy subjects during a response inhibition task. Theta oscillations are strongly modulated by correlated inhibitory control processes^43^; however, a strong correlation between pupil and theta oscillations is only seen in cases where inhibition of control processes is rarely required. This implies that the NE system modulates inhibitory control processes through theta band activity in the superior frontal gyrus when the likelihood of inhibiting a prepotent response tendency is low. Besides the NE system, the inhibitory neurotransmitter GABA is thought to contribute to the improvement of signal‐to‐noise ratio and inhibitory control,^56^ and also plays an important regulatory role in WM tasks and PFC neural oscillations.^57^ Increased striatal GABA levels were associated with better response inhibition and were predictive of the phase‐locking factor in the frontal midline theta‐related Nogo‐N2 time window.^58^

The potential mechanisms by which taVNS improves cognition are closely related to NE and GABA levels.^59^, ^60^, ^61^ Specifically, in taVNS treatment, stimulation is transmitted via vagal afferent fibers to the NTS, which directly or indirectly projects to various brain regions such as the locus coeruleus (LC), dorsal raphe nucleus (DRN), amygdala, hippocampus, PFC, and motor cortex, and regulates free NE and GABA levels in the brain by activating the LC‐NE system and NTS‐GABA system.^60^, ^61^ tVNS‐induced simultaneous modulation of the GABAergic and NA systems induced better response inhibition performance during WM.^9^ Appropriate levels of NE enhance PFC function by activating frontal postsynaptic α2‐receptors and act on hippocampal β‐receptors to promote synaptic plasticity by facilitating long‐term potentiation (LTP) in the hippocampal dentate gyrus, thereby enhancing cognitive performance.^62^, ^63^ On the other hand, chronic VNS may have a neuroprotective effect by promoting an increase in neurotrophic factor and NE release, reversing the reduced LC‐NE levels and leading to improved cognition.^64^ VNS has been demonstrated to activate NTS to increase the release of GABA.^65^ A treatment of tVNS at 0.5 mA enhanced divergent thinking in healthy subjects and explained the positive effect of tVNS on divergent thinking by increasing GABA concentration.^66^ In patients with epilepsy treated with VNS for 1 year, a significant increase in hippocampal GABA‐A receptor density was found to modulate cortical excitability and plasticity.^67^

Our results showed that prefrontal theta PSD during WM encoding, maintenance, and retrieval phases was reduced after 20 weeks of taVNS treatment, and WM behavior performance was improved. The mechanism by which taVNS modulates PFC theta oscillations may be related to the regulation of NE and GABAergic systems by taVNS. Diminished peak frontal NoGo N2 amplitude was found to be closely related to FMT in healthy subjects under tVNS, demonstrating that fewer cognitive control resources are required for inhibitory control of prepotent responses after tVNS.^10^ Anodal tDCS to the left dorsolateral PFC significantly reduced RT and N200 amplitude, which was used as a measure of attentional effort in subjects performing the state‐dependent Flanker task.^68^ These results imply a better signal‐to‐noise ratio and easier and more efficient use of available cognitive resources for signal detection, suggesting the optimization of cognitive control by tDCS. Anodal tDCS of 2 mA in the superior frontal gyrus during inhibitory control eliminated the positive time‐course correlation between pupil diameter and theta activity, suggesting that anodal tDCS eliminates NE‐mediated modulation of theta band activity through a gain control process modulated by the NE system and tDCS.^55^ In the current study, taVNS may modulate FMT with the NE and GABA systems, and future studies will examine the underlying neurophysiologic mechanisms.

The current study revealed alterations in neural oscillations during WM encoding, maintenance, and retrieval phases after chronic taVNS treatment (20 weeks) in rTLE patients. However, there were limitations to this study that should be noted. First, we examined only theta oscillations and additional studies are needed to determine the influence of taVNS on other frequency bands. Second, it was limited to the PFC, but neural oscillatory patterns in the hippocampus, sensory cortex, striatum, and other structures closely related to WM are also important and will be investigated in future research.

## AUTHOR CONTRIBUTIONS

All authors have made substantial contributions to the design and concept of this study. Yijun Song and Liping Pan designed the study, and Liping Pan, Yunfan Wang, and Yujing Zhu were involved in data collection. Liping Pan, Jiajing Wang, and Wenjuan Wu were involved in data analyses under the supervision of Yijun Song. Liping Pan and Jiajing Wang drafted the paper under the supervision of Yijun Song. All authors critically revised the manuscript. All authors read and approved the final manuscript.

## CONFLICT OF INTEREST STATEMENT

The authors declare that they have no competing interests.

## Supporting information


Figure S1.

## Data Availability

The data generated from this study are available upon request from the corresponding authors.

## References

[1] Miller Ek LMBA . Working memory 2.0. Neuron. 2018;100(2):463‐475. 30359609 10.1016/j.neuron.2018.09.023 PMC8112390

[2] D'Esposito M , Postle BR . The cognitive neuroscience of working memory. Annu Rev Psychol. 2015;66(1):115‐142. 25251486 10.1146/annurev-psych-010814-015031 PMC4374359

[3] Ludyga S , Mücke M , Leuenberger R , et al. Behavioral and neurocognitive effects of judo training on working memory capacity in children with ADHD: a randomized controlled trial. NeuroImage: Clinical. 2022;36:103156. 35988343 10.1016/j.nicl.2022.103156 PMC9402389

[4] Bellgrove M . 22.3 working memory and reaction time variability mediate the relationship between polygenic risk and ADHD‐like traits: evidence from a general population sample. J Am Acad Child Adolesc Psychiatry. 2022;61(Supplement 10):S311. 10.1038/s41380-022-01775-5 PMC9763105 36151456

[5] Poole BJ , Phillips NL , Stewart E , Harris IM , Lah S . Working memory in pediatric epilepsy: a systematic review and meta‐analysis. Neuropsychol Rev. 2021;31(4):569‐609. 33818735 10.1007/s11065-021-09491-7

[6] Arski ON , Wong SM , Warsi NM , et al. Epilepsy disrupts hippocampal phase precision and impairs working memory. Epilepsia. 2022;63(10):2583‐2596. 35778973 10.1111/epi.17357

[7] Boon P , Vonck K , Vandekerckhove T , et al. Vagus nerve stimulation for medically refractory epilepsy; efficacy and cost‐benefit analysis. Acta Neurochir. 1999;141(5):447‐452. 10392199 10.1007/s007010050324

[8] Sun J , Cheng C , Tian Q , et al. Transcutaneous auricular vagus nerve stimulation improves spatial working memory in healthy young adults. Front Neurosci. 2021;15:15. 10.3389/fnins.2021.790793 PMC8733384 35002607

[9] Beste C , Steenbergen L , Sellaro R , et al. Effects of concomitant stimulation of the GABAergic and norepinephrine system on inhibitory control—a study using transcutaneous vagus nerve stimulation. Brain Stimul. 2016;9(6):811‐818. 27522167 10.1016/j.brs.2016.07.004

[10] Pihlaja M , Failla L , Peräkylä J , Hartikainen KM . Reduced frontal Nogo‐N2 with uncompromised response inhibition during transcutaneous vagus nerve stimulation—more efficient cognitive control? Front Hum Neurosci. 2020;14:561780. 33132877 10.3389/fnhum.2020.561780 PMC7573492

[11] Jacobs HI , Riphagen JM , Razat CM , Wiese S , Sack AT . Transcutaneous vagus nerve stimulation boosts associative memory in older individuals. Neurobiol Aging. 2015;36(5):1860‐1867. 25805212 10.1016/j.neurobiolaging.2015.02.023

[12] González HFJ , Yengo‐Kahn A , Englot DJ . Vagus nerve stimulation for the treatment of epilepsy. Neurosurg Clin N Am. 2019;30(2):219‐230. 30898273 10.1016/j.nec.2018.12.005 PMC6432928

[13] Stefan H , Kreiselmeyer G , Kerling F , et al. Transcutaneous vagus nerve stimulation (t‐VNS) in pharmacoresistant epilepsies: a proof of concept trial. Epilepsia. 2012;53(7):e115‐e118. 22554199 10.1111/j.1528-1167.2012.03492.x

[14] Mertens A , Gadeyne S , Lescrauwaet E , et al. The potential of invasive and non‐invasive vagus nerve stimulation to improve verbal memory performance in epilepsy patients. Sci Rep. 2022;12(1):1984. 35132096 10.1038/s41598-022-05842-3 PMC8821667

[15] de Vries IEJ , Slagter HA , Olivers CNL . Oscillatory control over representational states in working memory. Trends Cogn Sci. 2020;24(2):150‐162. 31791896 10.1016/j.tics.2019.11.006

[16] Sauseng P , Griesmayr B , Freunberger R , Klimesch W . Control mechanisms in working memory: a possible function of EEG theta oscillations. Neurosci Biobehav Rev. 2010;34(7):1015‐1022. 20006645 10.1016/j.neubiorev.2009.12.006

[17] Pan L , Guo D , Wang J , et al. Alterations in neural oscillations related to working memory deficit in temporal lobe epilepsy. Epilepsy Behav. 2021;121:108063. 34052633 10.1016/j.yebeh.2021.108063

[18] Sun L , Peräkylä J , Holm K , et al. Vagus nerve stimulation improves working memory performance. J Clin Exp Neuropsychol. 2017;39(10):954‐964. 28492363 10.1080/13803395.2017.1285869

[19] Stretton J , Winston GP , Sidhu M , et al. Disrupted segregation of working memory networks in temporal lobe epilepsy. NeuroImage: Clinical. 2013;2:273‐281. 24179782 10.1016/j.nicl.2013.01.009 PMC3777779

[20] Pan L , Wu Y , Bao J , et al. Alterations in neural networks during working memory encoding related to cognitive impairment in temporal lobe epilepsy. Front Hum Neurosci. 2021;15:770678. 35069151 10.3389/fnhum.2021.770678 PMC8766724

[21] Löscher W , Potschka H , Sisodiya SM , Vezzani A . Drug resistance in epilepsy: clinical impact, potential mechanisms, and new innovative treatment options. Pharmacol Rev. 2020;72(3):606‐638. 32540959 10.1124/pr.120.019539 PMC7300324

[22] Kwan P , Arzimanoglou A , Berg AT , et al. Definition of drug resistant epilepsy: consensus proposal by the ad hoc Task Force of the ILAE Commission on Therapeutic Strategies. Epilepsia. 2010;51(6):1069‐1077. 19889013 10.1111/j.1528-1167.2009.02397.x

[23] Badran BW , Brown JC , Dowdle LT , et al. Tragus or cymba conchae? Investigating the anatomical foundation of transcutaneous auricular vagus nerve stimulation (taVNS). Brain Stimul. 2018;11(4):947‐948. 29895444 10.1016/j.brs.2018.06.003 PMC6607436

[24] Rossion B , Pourtois G . Revisiting Snodgrass and Vanderwart's object pictorial set: the role of surface detail in basic‐level object recognition. Perception. 2004;33(2):217‐236. 15109163 10.1068/p5117

[25] Zhang D , Zhao H , Bai W , Tian X . Functional connectivity among multi‐channel EEGs when working memory load reaches the capacity. Brain Res. 2016;1631:101‐112. 26638838 10.1016/j.brainres.2015.11.036

[26] Hsieh L , Ranganath C . Frontal midline theta oscillations during working memory maintenance and episodic encoding and retrieval. Neuroimage. 2014;85:721‐729. 23933041 10.1016/j.neuroimage.2013.08.003 PMC3859771

[27] Mitchell DJ , McNaughton N , Flanagan D , Kirk IJ . Frontal‐midline theta from the perspective of hippocampal “theta”. Prog Neurobiol. 2008;86(3):156‐185. 18824212 10.1016/j.pneurobio.2008.09.005

[28] Murphy OW , Hoy KE , Wong D , Bailey NW , Fitzgerald PB , Segrave RA . Individuals with depression display abnormal modulation of neural oscillatory activity during working memory encoding and maintenance. Biol Psychol. 2019;148:107766. 31509766 10.1016/j.biopsycho.2019.107766

[29] Kardos Z , Tóth B , Boha R , File B , Molnár M . Age‐related changes of frontal‐midline theta is predictive of efficient memory maintenance. Neuroscience. 2014;25:152‐162. 10.1016/j.neuroscience.2014.04.071 24846615

[30] Meyer L , Grigutsch M , Schmuck N , Gaston P , Friederici AD . Frontal‐posterior theta oscillations reflect memory retrieval during sentence comprehension. Cortex. 2015;71:205‐218. 26233521 10.1016/j.cortex.2015.06.027

[31] Onton J , Delorme A , Makeig S . Frontal midline EEG dynamics during working memory. Neuroimage. 2005;27(2):341‐356. 15927487 10.1016/j.neuroimage.2005.04.014

[32] Roux F , Uhlhaas PJ . Working memory and neural oscillations: alpha–gamma versus theta–gamma codes for distinct WM information? Trends Cogn Sci. 2014;18(1):16‐25. 24268290 10.1016/j.tics.2013.10.010

[33] Zielinski MC , Tang W , Jadhav SP . The role of replay and theta sequences in mediating hippocampal‐prefrontal interactions for memory and cognition. Hippocampus. 2018;30(1):60‐72. 29251801 10.1002/hipo.22821 PMC6005707

[34] Yu S , Mückschel M , Rempel S , Ziemssen T , Beste C . Time‐on‐task effects on working memory gating processes—a role of theta synchronization and the norepinephrine system. Cerebral Cortex Commun. 2022;3(1):tgac001. 10.1093/texcom/tgac001 PMC8794645 35098128

[35] Lee H , Simpson GV , Logothetis NK , Rainer G . Phase locking of single neuron activity to theta oscillations during working memory in monkey extrastriate visual cortex. Neuron. 2005;45(1):147‐156. 15629709 10.1016/j.neuron.2004.12.025

[36] Liebe S , Hoerzer GM , Logothetis NK , Rainer G . Theta coupling between V4 and prefrontal cortex predicts visual short‐term memory performance. Nat Neurosci. 2012;15(3):456‐462. 22286175 10.1038/nn.3038

[37] Cavanagh JF , Frank MJ . Frontal theta as a mechanism for cognitive control. Trends Cogn Sci. 2014;18(8):414‐421. 24835663 10.1016/j.tics.2014.04.012 PMC4112145

[38] Jacobs J , Hwang G , Curran T , Kahana MJ . EEG oscillations and recognition memory: theta correlates of memory retrieval and decision making. Neuroimage. 2006;32(2):978‐987. 16843012 10.1016/j.neuroimage.2006.02.018

[39] Roberts BM , Hsieh L , Ranganath C . Oscillatory activity during maintenance of spatial and temporal information in working memory. Neuropsychologia. 2013;51(2):349‐357. 23084981 10.1016/j.neuropsychologia.2012.10.009 PMC3546228

[40] Hsieh LT , Ekstrom AD , Ranganath C . Neural oscillations associated with item and temporal order maintenance in working memory. J Neurosci. 2011;31(30):10803‐10810. 21795532 10.1523/JNEUROSCI.0828-11.2011 PMC3164584

[41] Lisman JE , Jensen O . The theta–gamma neural code. Neuron. 2013;77(6):1002‐1016. 23522038 10.1016/j.neuron.2013.03.007 PMC3648857

[42] Pavlov YG , Kotchoubey B . Oscillatory brain activity and maintenance of verbal and visual working memory: a systematic review. Psychophysiology. 2022;59(5):e13735. 33278030 10.1111/psyp.13735

[43] Dippel G , Mückschel M , Ziemssen T , Beste C . Demands on response inhibition processes determine modulations of theta band activity in superior frontal areas and correlations with pupillometry—implications for the norepinephrine system during inhibitory control. Neuroimage. 2017;157:575‐585. 28647483 10.1016/j.neuroimage.2017.06.037

[44] Ragland JD , Maddock RJ , Hurtado MY , et al. Disrupted GABAergic facilitation of working memory performance in people with schizophrenia. NeuroImage: Clinical. 2020;25:102127. 31864216 10.1016/j.nicl.2019.102127 PMC6928454

[45] Benchenane K , Tiesinga PH , Battaglia FP . Oscillations in the prefrontal cortex: a gateway to memory and attention. Curr Opin Neurobiol. 2011;21(3):475‐485. doi:10.1016/j.conb.2011.01.004 21429736

[46] Kurzban R , Duckworth A , Kable JW , Myers J . An opportunity cost model of subjective effort and task performance. Behav Brain Sci. 2013;36(6):661‐679. 24304775 10.1017/S0140525X12003196 PMC3856320

[47] Rac‐Lubashevsky R , Frank MJ . Analogous computations in working memory input, output and motor gating: electrophysiological and computational modeling evidence. PLoS Comput Biol. 2021;17(6):e1008971. 34097689 10.1371/journal.pcbi.1008971 PMC8211210

[48] Colgin LL . Oscillations and hippocampal–prefrontal synchrony. Curr Opin Neurobiol. 2011;21(3):467‐474. 21571522 10.1016/j.conb.2011.04.006 PMC3578407

[49] Sauseng P , Klimesch W , Heise KF , et al. Brain oscillatory substrates of visual short‐term memory capacity. Curr Biol. 2009;19(21):1846‐1852. 19913428 10.1016/j.cub.2009.08.062

[50] Fujisawa S , Buzsáki G . A 4 Hz oscillation adaptively synchronizes prefrontal, VTA, and hippocampal activities. Neuron (Cambridge, Mass.). 2011;72(1):153‐165. 10.1016/j.neuron.2011.08.018 PMC3235795 21982376

[51] Aston‐Jones G , Cohen JD . An integrative theory of locus coeruleus‐norepinephrine function: adaptive gain and optimal performance. Annu Rev Neurosci. 2005;28:403‐450. 16022602 10.1146/annurev.neuro.28.061604.135709

[52] Garcia‐Junco‐Clemente P , Tring E , Ringach DL , Trachtenberg JT . State‐dependent subnetworks of parvalbumin‐expressing interneurons in neocortex. Cell Rep. 2019;26(9):2282‐2288. 30811979 10.1016/j.celrep.2019.02.005 PMC6407626

[53] Bensmann W , Zink N , Arning L , Beste C , Stock A . The presynaptic regulation of dopamine and norepinephrine synthesis has dissociable effects on different kinds of cognitive conflicts. Mol Neurobiol. 2019;56(12):8087‐8100. 31183808 10.1007/s12035-019-01664-z

[54] Nieuwenhuis S , Aston‐Jones G , Cohen JD . Decision making, the P3, and the locus coeruleus‐norepinephrine system. Psychol Bull. 2005;131(4):510‐532. 16060800 10.1037/0033-2909.131.4.510

[55] Adelhöfer N , Mückschel M , Teufert B , Ziemssen T , Beste C . Anodal tDCS affects neuromodulatory effects of the norepinephrine system on superior frontal theta activity during response inhibition. Brain Struct Funct. 2019;224(3):1291‐1300. 30701308 10.1007/s00429-019-01839-3

[56] Leventhal AG , Wang Y , Pu M , Zhou Y , Ma Y . GABA and its agonists improved visual cortical function in senescent monkeys. Science. 2003;300(5620):812‐815. 12730605 10.1126/science.1082874

[57] Lett TA , Voineskos AN , Kennedy JL , Levine B , Daskalakis ZJ . Treating working memory deficits in schizophrenia: a review of the neurobiology. Biol Psychiatry. 2014;75(5):361‐370. 24011822 10.1016/j.biopsych.2013.07.026

[58] Quetscher C , Yildiz A , Dharmadhikari S , et al. Striatal GABA‐MRS predicts response inhibition performance and its cortical electrophysiological correlates. Brain Struct Funct. 2015;220(6):3555‐3564. 25156575 10.1007/s00429-014-0873-y PMC4447607

[59] Clancy JA , Deuchars SA , Deuchars J . The wonders of the Wanderer. Exp Physiol. 2013;98(1):38‐45. 22848084 10.1113/expphysiol.2012.064543

[60] Colzato L , Beste C . A literature review on the neurophysiological underpinnings and cognitive effects of transcutaneous vagus nerve stimulation: challenges and future directions. J Neurophysiol. 2020;123(5):1739‐1755. 32208895 10.1152/jn.00057.2020

[61] Kumaria A , Sitaraman M . Can vagus nerve stimulation improve social cognition in autism? Cortex. 2019;115:350‐351. 29615199 10.1016/j.cortex.2018.02.020

[62] Spencer‐Smith M , Klingberg T . Benefits of a working memory training program for inattention in daily life: a systematic review and meta‐analysis. PLoS ONE. 2015;10(3):e119522. 10.1371/journal.pone.0119522 PMC4368783 25793607

[63] Arnsten AFT . Catecholamine influences on dorsolateral prefrontal cortical networks. Biol Psychiatry. 2011;69(12):e89‐e99. 21489408 10.1016/j.biopsych.2011.01.027 PMC3145207

[64] Merrill CA , Jonsson MAG , Minthon L , et al. Vagus nerve stimulation in patients with Alzheimer's disease: additional follow‐up results of a pilot study through 1 year. J Clin Psychiatry. 2006;67(8):1171‐1178. 16965193 10.4088/jcp.v67n0801

[65] Yakunina N , Kim SS , Nam E . Optimization of transcutaneous vagus nerve stimulation using functional MRI. Neuromodulation: Technol Neural Interface. 2017;20(3):290‐300. 10.1111/ner.12541 27898202

[66] Colzato LS , Ritter SM , Steenbergen L . Transcutaneous vagus nerve stimulation (tVNS) enhances divergent thinking. Neuropsychologia. 2018;111:72‐76. 29326067 10.1016/j.neuropsychologia.2018.01.003

[67] Marrosu F , Serra A , Maleci A , Puligheddu M , Biggio G , Piga M . Correlation between GABA(A) receptor density and vagus nerve stimulation in individuals with drug‐resistant partial epilepsy. Epilepsy Res. 2003;55(1–2):59‐70. 12948617 10.1016/s0920-1211(03)00107-4

[68] Dubreuil‐Vall L , Chau P , Ruffini G , Widge AS , Camprodon JA . tDCS to the left DLPFC modulates cognitive and physiological correlates of executive function in a state‐dependent manner. Brain Stimul. 2019;12(6):1456‐1463. 31221553 10.1016/j.brs.2019.06.006 PMC6851462

