# Supplementary material for: Transcutaneous auricular vagus nerve stimulation improves working memory in temporal lobe epilepsy: A randomized double‐blind study
Source: CNS Neurosci Ther. 2023 Aug 8;30(2):e14395. doi: 10.1111/cns.14395 (PMC10848055; doi:10.1111/cns.14395)
Supplement: Supplementary file 1 — Figure S1. [file CNS-30-e14395-s001.docx]

**
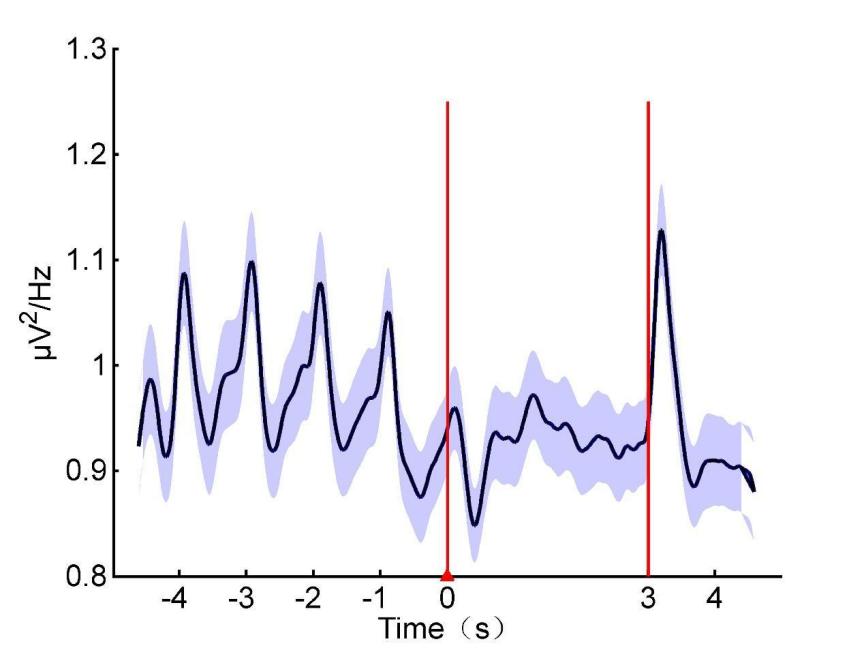
**

**Supplementary Fig. 1 Representative curve of theta power during the WM task**. The *x* axis indicates the time course of the task; the left, central, and right areas separated by the solid red lines represent the encoding, maintenance, and retrieval phases of WM, respectively. The *y* axis indicates the average theta PSD of 19 channels during the WM task. PSD, power spectral density.
